# Chemical and Genetic Screens Hit the Target in Cytokinesis

**DOI:** 10.1371/journal.pbio.0020386

**Published:** 2004-10-05

Cytokinesis, in which newly formed daughter cells separate, is the culmination of the cell cycle. It is necessary for normal growth and development, and it is also a sine qua non in the pathogenesis of cancers—cells that can't divide can't form tumors, can't metastasize, and can't kill. Therefore, understanding the full range of proteins involved in cytokinesis has both deep theoretical and immediate practical applications. In this issue, Ulrike Eggert and colleagues report results from two complementary screening approaches to identify those proteins and to discover molecules that inhibit them.

A cell cannot divide if it lacks a protein vital for cytokinesis or if that protein is inhibited. When that occurs, the cell retains both nuclei, and can be quickly identified by an automated process. Working with Drosophila cells, the first screen used almost 20,000 double-stranded RNAs, representing virtually the entire Drosophila genome. A double-stranded RNA pairs with, and causes the destruction of, a matching messenger RNA, thus preventing the encoded protein from being formed, a process called RNA interference (RNAi). The authors identified 214 proteins whose absence prevented cytokinesis. While some of these, including actin and Myosin, were already known to be essential for the process, others were not. The latter included a new discovery, CG4454 (named Borealin-related or Borr), which was found to be one of the handful of proteins deemed most critical to cytokinesis.[Fig pbio-0020386-g001]

**Figure pbio-0020386-g001:**
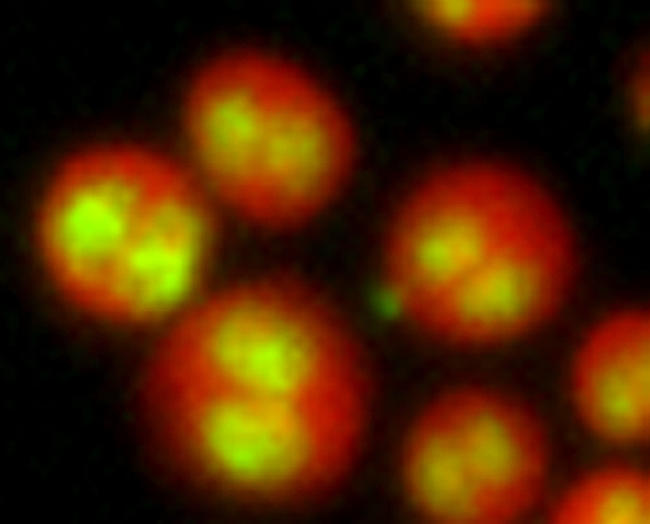
Drosophila cells that have failed to divide

The second screen also treated Drosophila cells, but this time used over 50,000 “small molecules,” a catchall term for molecules small enough to pass easily into cells. The vast majority of drugs currently in clinical use are small molecules. This screen revealed 50 cytokinesis inhibitors, of which 25, dubbed binucleines, were selected for further characterization. Not surprisingly, several inhibited actin, whose role in cytokinesis is key in contraction of the cytokinesis furrow.

For the purposes of this study, however, binucleines affecting other proteins were even more interesting. By comparing the appearances of binucleate cells from the small-molecule screen with those from the RNAi screen, Eggert et al. identified one molecule and three proteins that caused a similar phenotype, suggesting that the three proteins acted within a single pathway, which the molecule could disrupt. One of the three proteins was CG4454/Borr, and the researchers' results indicated it interacts with Aurora B, an essential but still poorly understood protein that is needed for proper division of the chromosomes. The identified binucleine will be a valuable reagent for exploring the details of the Aurora B pathway.

While these results are from Drosophila, the insights they provide into the cell cycle are likely to be applicable to humans as well. Equally importantly, they provide the proof of principle for a new drug discovery method. A major bottleneck in drug development is target identification—determining which of the cell's thousands of proteins is the right one to inhibit with a drug. The unique aspect of this study is the parallel use of the two approaches—small molecules and RNAi—to provide a “stereoscopic” view of cytokinesis and its inhibition. Working together, they provide a set of proteins and a matched set of inhibitors, the target and the bullet at the same time.

